# Supplementary material for: Mycotoxin Analysis of Grain via Dust Sampling: Review, Recent Advances and the Way Forward: The Contribution of the MycoKey Project
Source: Toxins (Basel). 2022 May 31;14(6):381. doi: 10.3390/toxins14060381 (PMC9227346; doi:10.3390/toxins14060381)
Supplement: Supplementary file 1 [file toxins-14-00381-s001.zip › toxins-1740767-supplementary.pdf]

toxins MDPI

Supplementary Materials: Mycotoxin Analysis of Grain via Dust Sampling: Review, Recent Advances and the Way Forward: the Contribution of the MycoKey Project

Biancamaria Ciasca, Sarah De Saeger, Marthe De Boevre, Mareike Reichel, Michelangelo Pascale, Antonio F. Logrieco and Veronica M. T. Lattanzio

Table S1. Parameters used for data acquisition in selected reaction monitoring (SRM).

Compound	Target m/z	Target ion	Cone Voltage (V)	Fragment ions	Collision energy (eV)	RT (min)
NIV	313	[M+H]+	35	177	21	4.2
				175*	16	
DON	297	[M+H]+	40	231	9	4.9
				249*	9	
AFB1	313.1	[M+H]+	65	241	32	7.5
				285	20	
HT-2	442.3	[M+NH4]+	40	215*	12	8.2
				263	12	
FB1	722.4	[M+H]+	40	334	36	8.3
				352*	32	
T-2	484.3	[M+NH4]+	40	215*	18	8.7
				305	12	
ZEN	319.2	[M+H]+	40	187	18	9.2
				283*	12	
OTA	404.0	[M+H]+	30	221	30	9.4
				240*	20	
FB2	706.1	[M+H]+	70	354	30	9.2
				336*	36	

* Transition used for analyte quantification, retention time (RT), volt (V), electronvolt (eV).

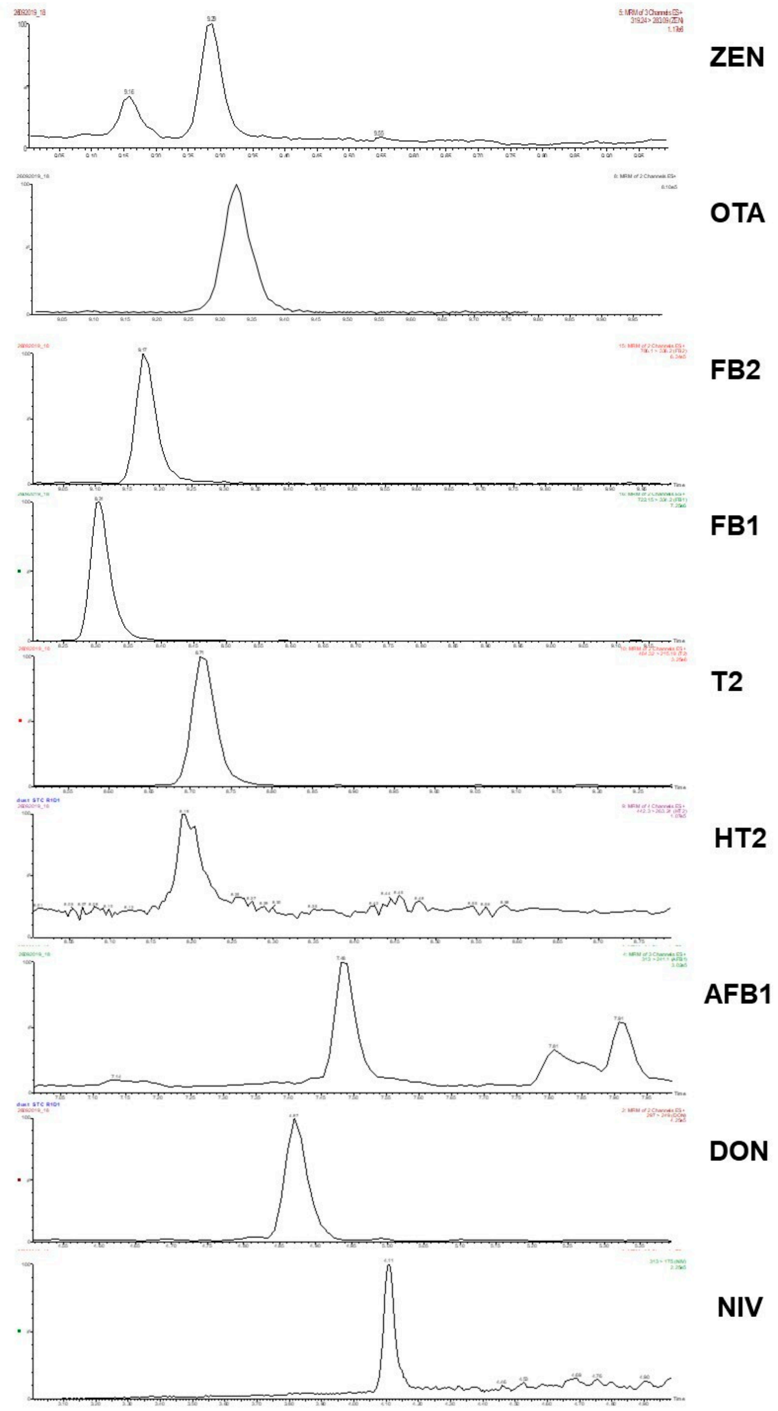

Figure S1. Selected ion chromatogram (quantifier SRM – select ion monitoring transition) of a dust sample extract spiked with nivalenol (NIV), deoxinivalenol (DON), Fuminisin B1 (FB1), Fumonisin B2 (FB2) 1000 µg/kg; HT2 toxin (HT2) and T-2 toxin (T2) 250 µg/kg; zeralenon (ZEN) 500 µg/kg, ochratoxin A (OTA) 15 µg/kg; aflatoxin B1 (AFB1) 10 µg/kg. .

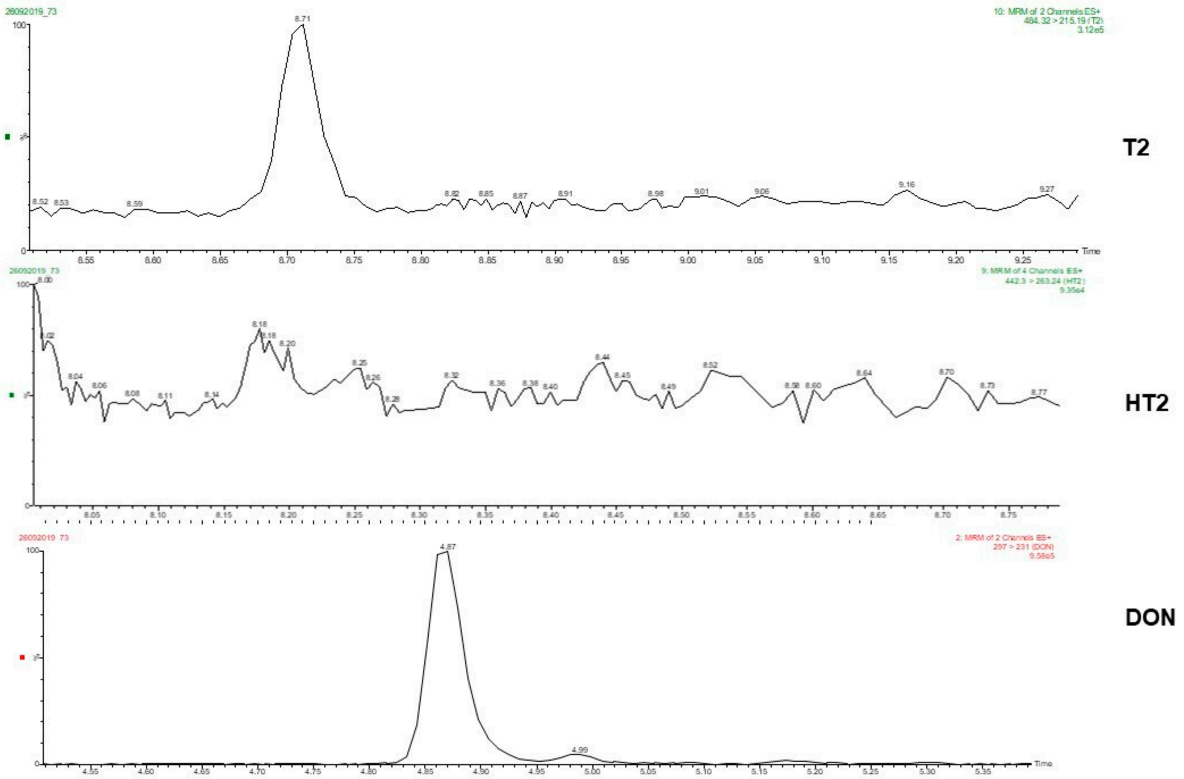

Figure S2. : Selected ion chromatogram (quantifier SRM – select ion monitoring transition) of a dust sample extract naturally contaminated with deoxinivalenol (DON) 7282 µg/kg,T-2 toxin (T2) 16 µg/kg and trace of HT-2 toxin (HT2) (< LOQ).

Table S2. In-house analytical performances of the LC-MS/MS method for multimycotoxins method in grains and dust, including spiking levels set at 25% of target level (µg/kg) and 100% of target level, mean recovery %,and the total relative standard deviation of the measurements - intermediate precision (RSDip, %). Mean recovery and RSDip,% are calculatedfrom the results of 12 experiments (three independent replicates for 4 daysover a time period of 1 year).

GRAINS (maize/wheat)				
	25% of target level (µg/kg)	Mean Recovery, % (RSDIP, %)* (n=12)	100% of target level (µg/kg)	Mean Recovery, % % (RSDIP, %)* (n=12)
**NIV**	250	102 (16)	1000	95 (15)
**DON**	250	95 (21)	1000	91 (19)
**AFB_1**	2.5	99 (15)	10	98 (7)
**HT2**	12.5	114 (26)	50	102 (11)
**T2**	12.5	110 (6)	50	100 (11)
**FB1**	250	110 (10)	1000	106 (27)
**FB2**	250	104 (19)	1000	107 (9)
**OTA**	2	125 (13)	8	125 (13)
**ZEN**	25	89 (22)	100	111 (23)
DUST (maize/wheat)				
	25% of target level (µg/kg)	Mean Recovery, % % (RSDIP, %)*	100% of target level (µg/kg)	Mean Recovery, % (RSDIP, %)*
**NIV**	250	80 (20)	1000	101 (22)
**DON**	250	91 (21)	1000	100 (32)
**AFB_1**	2.5	88 (13)	10	88 (16)

**HT2**	62.5	78 (19)	250	89 (12)
**T2**	62.5	86 (15)	250	87 (21)
**FB1**	250	95 (9)	1000	96 (1)
**FB2**	250	101 (10)	1000	101 (16)
**OTA**	3.7	84 (25)	15	103 (12)
**ZEN**	125	77 (25)	500	81 (16)

Table S3. Expanded uncertainty (U), at 95% of confidence level, calculated using the Horwitz equation.

	U (μg/kg)									
	NIV		DON		T2+HT2		ZEN		OTA	
	Grain	Dust	Grain	Dust	Grain	Dust	Grain	Dust	Grain	Dust
Trial 1										
Sample set 1										
Wagon 1	(-)	118	(-)	346	(-)	137	(-)	(-)	(-)	(-)
Wagon 2	(-)	73	(-)	335	(-)	114	(-)	(-)	(-)	(-)
Wagon 3	(-)	67	(-)	193	(-)	115	(-)	(-)	(-)	(-)
Wagon 4	(-)	43	(-)	175	(-)	80	(-)	(-)	(-)	(-)
Wagon 5	(-)	101	(-)	282	(-)	225	(-)	(-)	(-)	(-)
Wagon 6	(-)	68	(-)	232	(-)	137	(-)	(-)	(-)	(-)
Trial 2										
Sample set 2A										
truck 1	(-)	39	(-)	581	(-)	12	(-)	26	(-)	(-)
truck 2	(-)	51	(-)	263	(-)	6	(-)	(-)	(-)	2
truck 3	(-)	36	20	732	(-)	35	(-)	(-)	(-)	(-)
truck 4	(-)	37	34	982	(-)	7	(-)	118	(-)	26
truck 5 A	(-)	22	24	389	(-)	9	(-)	(-)	(-)	(-)
truck 6 A	(-)	68	35	1157	(-)	2	(-)	(-)	(-)	0
truck 7 A	(-)	129	(-)	355	(-)	11	(-)	(-)	(-)	(-)
truck 8	(-)	83	(-)	346	52	144	(-)	(-)	(-)	(-)
truck 9	(-)	28	(-)	381	(-)	9	(-)	(-)	(-)	(-)
truck 10	(-)	9	(-)	132	(-)	3	(-)	(-)	(-)	4
truck 11 A	(-)	100	(-)	372	9.5	121	(-)	(-)	(-)	(-)
truck 12	(-)	(-)	(-)	1084	(-)	4	(-)	(-)	(-)	(-)
truck 13	(-)	42	(-)	364	54	135	(-)	(-)	(-)	(-)
truck 14	(-)	72	23	995	(-)	3	(-)	(-)	(-)	(-)
truck 15 A	(-)	43	(-)	619	(-)	6	(-)	(-)	(-)	(-)
truck 16 A	(-)	76	36	1741	(-)	9	(-)	(-)	(-)	(-)
truck 17	(-)	98	(-)	1276	4	78	(-)	37	(-)	4
truck 18	(-)	134	(-)	1119	(-)	161	(-)	(-)	(-)	(-)
truck 19 A	(-)	72	15	1521	(-)	41	(-)	108	(-)	9
Truck 20	(-)	235	(-)	484	3	78	(-)	(-)	(-)	1
truck 21	(-)	126	9	807	14	135	(-)	(-)	(-)	(-)
truck 22	(-)	78	(-)	944	(-)	79	(-)	(-)	(-)	2
truck 23	(-)	70	30	1341	(-)	7	(-)	(-)	(-)	(-)
truck 24	(-)	156	9	722	(-)	10	(-)	(-)	(-)	0
truck 25	(-)	146	11	820	11	130	(-)	(-)	(-)	(-)
truck 26	(-)	84	(-)	637	(-)	134	(-)	(-)	(-)	(-)
truck 27	(-)	103	(-)	375	5	280	(-)	(-)	(-)	(-)

truck 28 A	(-)	98	(-)	392	8	209	(-)	(-)	(-)	1
truck 29	(-)	125	11	442	12	19	(-)	92	(-)	1
truck 30	(-)	77	10	517	21	143	(-)	(-)	(-)	(-)
truck 31	(-)	114	40	2364	5	65	(-)	(-)	(-)	2
truck 32	(-)	109	14	1034	(-)	(-)	(-)	(-)	(-)	(-)
Sample set 2B										
truck 5 B	(-)	69	11	363	(-)	34	(-)	(-)	(-)	(-)
truck 6 B	(-)	76	15	1292	(-)	2	(-)	(-)	(-)	(-)
truck 7 B	(-)	196	(-)	728	(-)	81	(-)	(-)	(-)	(-)
truck 11 B	(-)	112	(-)	301	4	117	(-)	(-)	(-)	(-)
truck 15 B	(-)	46	(-)	729	(-)	7	(-)	(-)	(-)	(-)
truck 16 B	(-)	94	16	1728	(-)	10	(-)	(-)	(-)	(-)
truck 19 B	(-)	(-)	15	1584	(-)	(-)	(-)	(-)	(-)	(-)
truck 28 B	(-)	(-)	(-)	276	8	(-)	(-)	(-)	(-)	(-)
